# Antimicrobial resistance profile of extended-spectrum beta-lactamases, adenosine-monophosphate-cyclic, and carbapenemase-producing Gram-negative bacteria isolated from domestic animals

**DOI:** 10.14202/vetworld.2021.3099-3104

**Published:** 2021-12-13

**Authors:** Jôiciglecia Pereira dos Santos, Valesca Ferreira Machado de Souza, Marcos Wilker da Conceição Santos, Juliany Nunes dos Santos, Natilene Silva dos Santos, Angélica Prado de Oliveira, Valquíria Tatiele da Silva Rodrigues, Ianei Oliveira Carneiro, Layze Cilmara Alves da Silva Vieira

**Affiliations:** 1 Campus of Agrarian Sciences, Federal University of Vale do São Francisco - UNIVASF, Petrolina, PE, Brazil; 2 Centro Multidisciplinar Campus Barra, Federal University of Western Bahia - UFOB, Barra, BA, Brazil; 3 Salvador University (UNIFACS), Salvador, BA, Brazil

**Keywords:** antimicrobial resistance, extended-spectrum beta-lactamase, *Klebsiella pneumoniae* carbapenemase, phenotypic tests

## Abstract

**Background and Aim:**

The production of beta-lactamase enzymes, such as extended-spectrum beta-lactamase (ESBL), adenosine-monophosphate-cyclic (AmpC), and *Klebsiella pneumoniae* carbapenemase (KPC), is one of the most important mechanisms of bacterial resistance to antimicrobials. Gram-negative bacteria show significant resistance due to various intrinsic and acquired factors. These intrinsic factors include low permeability of the outer membrane, various efflux systems, and the production of beta-lactamases, while acquired factors include chromosomal mutation and acquisition of resistance genes by horizontal transfer. Mobile elements such as plasmids, integrative conjugative elements, mobilizable islands, or transposable elements are involved in horizontal transfer. At present, the Gram-negative pathogens of most concern are *Acinetobacter baumannii*, *Pseudomonas aeruginosa*, and those belonging to the *Enterobacteriaceae* family (e.g., *Escherichia coli*, *K. pneumoniae*, and *Proteus mirabilis*). This study aimed to evaluate the profile of antimicrobial resistance and the production of the enzymes ESBL, AmpC, and KPC, in 21 gram-negative bacteria isolated from domestic animals treated at the University Veterinary Hospital (HVU) of the Federal University of Western Bahia (UFOB).

**Materials and Methods:**

The biological samples (21) were inoculated to brain heart infusion broth, blood agar, and MacConkey agar and incubated for 24-72 h at 37°C. Gram staining and identification through biochemical tests and matrix-associated laser desorption/ionization time-of-flight mass spectrometry were conducted. To evaluate the antimicrobial resistance profile, the disk diffusion method was used, and 25 antibiotics were employed. For the detection of ESBL, the disk approximation method was applied using chromogenic agar. The presence of KPC was observed using chromogenic agar and the Hodge test. For AmpC evaluation, the disk approximation method was used.

**Results:**

The most isolated agent was *E. coli* (66.66%, 14/21), followed by *K. pneumoniae* and *P. mirabilis* (both 14.29%, 3/21), and then *Pasteurella* spp. (4.76%, 1/21). The bacterial isolates showed high levels of resistance against clindamycin, penicillin, imipenem, polymyxin, cefoxitin, gentamycin, cefotaxime, ceftazidime, cephalothin, ceftriaxone, ciprofloxacin, trimethoprim/sulfamethoxazole, chloramphenicol, and tetracycline. The best effectiveness rates were observed for cefepime, streptomycin, amoxicillin-clavulanate, aztreonam, nalidixic acid, tobramycin, levofloxacin, amikacin, and meropenem. All biological isolates showed multiple resistance to at least three of the antibiotics tested (3/25), and some showed resistance to 24 of the antibiotics tested (24/25). Among the 21 pathogens analyzed, 8 were ESBL producers (38.09%); of these, 6 were identified as *E. coli* (28.57%), and 2 were identified as *K. pneumoniae* (9.52%). Two strains of *K. pneumoniae* produced both ESBL and KPC. None of the isolates were producers of AmpC.

**Conclusion:**

The results found in the present work raise concern about the level of antimicrobial resistance among pathogens isolated from domestic animals in Brazil. The results highlight the need for the development and implementation of anti-resistance strategies to avoid the dissemination of multiresistant pathogens, including the prudent use of antimicrobials and the implementation of bacterial culture, antimicrobial sensitivity, and phenotypic tests for the detection of beta-lactamase enzymes in bacteria isolated from animals.

## Introduction

At present, one of the greatest threats to One Health is the bacterial resistance to antimicrobials [1-4]. This is a phenomenon that can occur naturally due to the intrinsic or acquired mechanisms of resistance, including mutations in target genes [3,4]. Several mechanisms make bacteria resistant to antibiotics, and when referring to beta-lactam antibiotics, one of the most important resistance mechanisms is the production of beta-lactamase enzymes, such as extended-spectrum beta-lactamases (ESBLs), adenosine-monophosphate-cyclic (AmpC)-type beta-lactamases, and carbapenemases, including *Klebsiella pneumoniae* carbapenemase (KPC). ESBLs are plasmid-mediated enzymes that hydrolyze the beta-lactam ring of penicillins, cephalosporins, and other antimicrobials, rendering them inactive. ESBLs and are inhibited by clavulanic acid, sulbactam, and tazobactam [1,5-8]. AmpC-type beta-lactamases are plasmid and/or chromosomal enzymes that can hydrolyze penicillins and up to third-generation cephalosporins, cephamycins, and monobactams. They are not inactivated by clavulanic acid or tazobactam, but are inhibited by carbapenems and avibactam [1,5,7,9]. Carbapenemases are beta-lactamases that are encoded by chromosomal and plasmid genes that hydrolyze carbapenem antibiotics, penicillins, cephalosporins, and aztreonam. They also hydrolyze clavulanic acid, sulbactam, and tazobactam. The only antimicrobial capable of inhibiting them is avibactam, but hydrolysis occurs in a slower manner. The carbapenemases that are commonly associated with *Enterobacteriaceae* are those belonging to the KPC family and Ambler Class A serine beta-lactamases [1,5,10,11].

Gram-negative bacteria are microorganisms which stand out with regard to resistance due to intrinsic and chromosomal factors inherent in some species. Among the beta-lactamase-producing Gram-negative bacteria, the most worrisome are *Acinetobacter baumannii*, *Pseudomonas aeruginosa*, and those of the *Enterobacteriaceae* family (e.g., *Escherichia coli, K. pneumoniae*, and *Proteus mirabilis*) [8,10,12-14]. Among the factors that have increased the rate of antibiotic resistance in recent years, their indiscriminate, erroneous, and/or abusive use in both human and veterinary medicine have been highlighted as well as the failure to perform bacterial culture, antimicrobial sensitivity, and phenotypic testing [3,10,12,15]. In this context, the role of veterinarians has gained increasing prominence because the prudent use of antimicrobials is part of their competence, since more than half of the antibiotics produced worldwide are used in both production and companion animals [2,13-16].

In view of this problem, this study aimed to evaluate the antimicrobial resistance profile and the production of ESBL, AmpC, and KPC in bacteria isolated from domestic animals with different diseases treated at the University Veterinary Hospital (HVU) of the Federal University of Western Bahia (UFOB), Barra Multidisciplinary Center.

## Materials and Methods

### Ethical approval

The study was approved by the Ethics Committee on Animal Use of the UFOB and received protocol number 00172019.

### Study period, location, and sample collection

The samples originate from domestic animals with various health conditions, with no predilection for age or gender, consulted from May 2018 to December 2019 at the UFOB’s HVU, Barra Multidisciplinary Center. The samples were collected using sterile swabs (Absorve^®^, Cral Laboratory Supplies Ltd., Brazil) and Stuart’s medium (HiMedia M306^®^, Dsyslab, Brazil) and packed in isothermal boxes with recyclable ice and sent for analysis at the Veterinary Microbiology Laboratory of the same institution.

### Isolation and identification of bacteria

For the bacterial isolation, biological samples were inoculated to brain heart infusion broth (BHI) (HiMedia M210^®^, Dsyslab), 5% sheep blood agar (HiMedia M073^®^, Dsyslab), and MacConkey agar (HiMedia M081^®^, Dsyslab), and incubated at 37°C in aerobiosis for 24-48 h. During the readings, the turbidity of the liquid medium and the morphological characteristics of the colonies on the solid medium were observed. Subsequently, morphotintorial analysis of the bacteria was performed using the Gram staining method. The bacteria were identified using biochemical tests based on Bergey’s Manual of Microbiology [11]. For greater reliability of the results, confirmation of the bacterial species was performed using matrix-associated laser desorption/ionization-time-of-flight (MALDI-TOF) mass spectrometry [17].

### *In vitro* antimicrobial resistance profiles

To evaluate the antimicrobial resistance profiles of each isolate, the disk diffusion method, proposed by the Clinical and Laboratory Standards Institute (CLSI) [18], was used. A total of 25 antibiotics were tested: Amoxicillin-clavulanate (20-10 μg), ampicillin (10 μg), amikacin (30 μg), aztreonam (30 μg), cephalexin (30 μg), ciprofloxacin (5 μg), enrofloxacin (5 μg), chloramphenicol (30 μg), streptomycin (10 μg), gentamicin (10 μg), trimethoprim/sulfamethoxazole (25 μg), tetracycline (30 μg), polymyxin (300 μg), cephalothin (30 μg), cefepime (30 μg), ceftazidime (30 μg), cefotaxime (30 μg), ceftriaxone (30 μg), imipenem (10 μg), azithromycin, (15 μg), nalidixic acid (30 μg), meropenem (10 μg), cefaclor (30 μg), tobramycin (10 μg), and cefoxitin (30 μg) (Laborclin, Brazil). For quality control, the American Type Culture Collection (ATCC) standard strain, *E. coli* no. 25922 was used.

### ESBL and KPC detection in chromogenic agar

The plates to be used were placed for 30 min in a 35°C bacteriological oven for thermal adjustment before use. Then, using a sterile microbiological loop, the streaking technique was carried out. Plates were incubated at 35°C for 24 h. After the incubation period, the colonies were observed, and the reading of the test was performed according to the manufacturer’s guidelines (Laborclin).

### KPC detection-Hodge test

To perform carbapenemase confirmatory tests, an *E. coli* ATCC 25922 suspension, equivalent to 0.5 McFarland scale, was seeded on Mueller-Hinton agar (MHA) (HiMedia M173^®^, Dsyslab). Then, a 10 μg imipenem disk (Laborclin) was placed in the center of the plates. Three colonies were selected and seeded in a line, from the edge of the antibiotic disk to the edge of the plate, which was incubated at 37°C for 20 h. The interpretation of the results was based on the reference values established by the CLSI [19]. The reference strain *K. pneumoniae* 13883 from the ATCC was used as a negative control.

### ESBL and AmpC detection by the disk approximation method

The inoculums were diluted to the standard 0.5 McFarland turbidity scale, then streaked onto MHA plates (HiMedia M173^®^, Dsyslab) using a sterile swab (Absorve^®^). For the detection of ESBL-producing isolates, an amoxicillin-clavulanic acid (30 μg) disk was placed in the center of the plates and ceftazidime (30 μg), cefotaxime (30 μg), ceftriaxone (30 μg), and cefepime (30 μg) disks (Laborclin) were arranged 2.5 cm away from it. The detection of AmpC production was performed by arranging a ceftazidime (30 μg) disk 3 cm away from an imipenem (10 μg) disk (Laborclin). For the detection of AmpC production, the pathogens were diluted until the turbidity matched the 0.5 McFarland standard and were subsequently streaked onto MHA plates (HiMedia M173^®^, Dsyslab) with a sterile swab (Absorve^®^). Then, a disk of ceftazidime (30 μg) was arranged 3 cm away from a disk of imipenem (10 μg) (Laborclin). The plates were incubated in a bacteriological oven at 37°C for a period of 18-24 h. The appearance of a truncated junction zone between the two disks is suggestive of the presence of an Ambler’s Class C enzyme [7]. The results were interpreted based on the reference values established by CLSI [19].

## Results

During the course of this study, a total of 400 animals were examined at the HVU-UFOB. Forty-five animals presented alterations suggestive of infectious conditions. Samples were collected, and the growth of 21 Gram-negative bacteria was obtained. Based on biochemical tests and mass spectrometry (MALDI-TOF), it was found that 66.66% (14/21) of the bacteria were *E. coli*, 14.29% (3/21) were *K. pneumoniae*; 14.29% (3/21) were *P. mirabilis*, and 4.76% (1/21) were *Pasteurella* spp. (Table-1).

**Table 1:** Frequency of isolation of Gram-negative bacteria from 21 samples of domestic animals treated at the University Veterinary Hospital of the Federal University of Western Bahia, Barra Multidisciplinary Center-BA.

Agent	AF	RF (%)
*Escherichia coli*	14	66.66
*Klebsiella pneumoniae*	3	14.29
*Proteus mirabilis*	3	14.29
*Pasteurella* spp.	1	4.76
Total	21	100

AF=Absolute frequency; RF=Relative frequency

The results indicated the high prevalence of *E. coli* in the clinical samples studied. *E. coli* was the pathogen that triggers the most canine diseases (47.61%, 10/21), and was isolated from clinical conditions of gastrointestinal infections (28.57%, 6/21), otitis (9.53%, 2/21), and pyometra (9.53%, 2/21), and was also associated with sepsis (19.05%, 4/21) and death in swine. *K. pneumoniae* bacteria affected pigs, dogs, and cats, causing respiratory infections (4.76%, 1/21), skin infections (4.76%, 1/21), and urinary tract infections (4.76%, 1/21), respectively. *P. mirabilis* was identified in an abscess in a horse (4.76%, 1/21) and in a case of respiratory illness in 2 dogs (9.53%, 2/21). *Pasteurella* spp. strain was isolated from only one dog and caused pyometra (4.76%, 1/21) (Table-2).

**Table 2:** Identification of Gram-negative bacteria according to the medical condition and animal species affected. Analysis performed using 21 biological samples from domestic animals treated at the University Veterinary Hospital of the Federal University of Western Bahia, Barra Multidisciplinary Center-BA.

Bacteria	Medical conditions	Animal species affected	Percentage (n)
*Escherichia coli*	Septicemia	Swine	19.05 (4)
	Gastrointestinal infection	Canine	28.57 (6)
	Pyometra	Canine	9.53 (2)
	Otitis	Canine	9.53 (2)
*Klebsiella pneumoniae*	Respiratory infection	Swine	4.76 (1)
	Pyodermatitis	Canine	4.76 (1)
	Urinary tract infection	Feline	4.76 (1)
*Pasteurella* spp.	Pyometra	Canine	4.76 (1)
*Proteus mirabilis*	Abscess with purulent secretion	Equine	4.76 (1)
	Respiratory infection	Canine	9.53 (2)
Total			100 (21)

Regarding the evaluation of antimicrobial susceptibility, it was noted that the bacteria showed a high resistance percentage to the different antibiotics tested (Table-3). All bacteria analyzed in this study showed multiple resistance to at least 3 (3/25) of the antimicrobials tested, and two bacteria showed resistance to 24 of the antimicrobials tested (24/25). Of the 21 bacteria analyzed, two, which were identified as *E. coli*, were resistant to 24 of the 25 antibiotics tested. Overall, most pathogens (11/21) were resistant to at least 13 of the antimicrobials. However, *Pasteurella* spp. showed low resistance rates and was resistant to only three of the tested drugs (Table-3).

**Table 3:** Antimicrobial resistance profile of *Escherichia coli, Klebsiella pneumoniae, Proteus mirabilis,* and *Pasteurella* spp., isolated from medical conditions of domestic animal treated at the University Veterinary Hospital of the Federal University of Western Bahia, Barra Multidisciplinary Center-BA.

Antibiotics	Bacterial resistance profile % *			

	*Escherichia coli*	*Klebsiella pneumoniae*	*Pasteurella* spp.	*Proteus mirabilis*
Clindamycin	100.00	100.00	100.00	100.00
Penicillin	100.00	100.00	100.00	100.00
Imipenem	78.57	100.00	100.00	66.67
Polymyxin	85.71	100.00	0.00	100.00
Cefoxitin	92.85	100.00	0.00	66.67
Gentamicin	92.85	100.00	0.00	66.67
Cefotaxime	78.57	100.00	0.00	66.67
Ceftazidime	71.42	100.00	0.00	66.67
Cephalothin	100.00	66.67	0.00	66.67
Ceftriaxone	85.71	66.67	0.00	66.67
Ciprofloxacin	50.00	100.00	0.00	66.67
Trimethoprim/sulfamethoxazole	50.00	100.00	0.00	66.67
Chloramphenicol	57.14	66.67	0.00	66.67
Tetracycline	42.85	66.67	0.00	66.67
Ampicillin	64.28	100.00	0.00	0.00
Cefaclor	57.14	33.33	0.00	66.67
Meropenem	21.42	66.67	0.00	66.67
Amikacin	50.00	0.00	0.00	100.00
Levofloxacin	42.85	33.33	0.00	66.67
Tobramycin	42.85	33.33	0.00	66.67
Nalidixic acid	35.71	66.67	0.00	0.00
Aztreonam	28.57	33.33	0.00	0.00
Amoxicillin+Clavulanate	57.14	0.00	0.00	0.00
Cefepime	35.71	0.00	0.00	0.00
Streptomycin	35.71	0.00	0.00	0.00

* Average per species

The best antimicrobial effectiveness rates were observed for cefepime, streptomycin, amoxicillin-clavulanate, aztreonam, nalidixic acid, tobramycin, levofloxacin, amikacin, and meropenem (Table-3).

Of the 21 bacteria analyzed, 38.09% (8/21) were ESBL producers. Of these, 6 (28.57%) were identified as *E. coli* and 2 (9.52%) were identified as *K. pneumoniae*. Out of the six ESBL-positive *E. coli*, 4 (19.05%) were identified in the canine species and 2 (9.52%) were identified in the swine species. In both the chromogenic agar and the Hodge test, two strains of *K. pneumoniae* were considered positive for KPC, and they had already been recognized as ESBL producers. The AmpC phenotype was not detected in this collection of isolates.

## Discussion

In the present study, infectious diseases were associated with environmental and opportunistic microorganisms, especially agents belonging to the family *Enterobacteriaceae*. It is believed that the ease of contact between the animals and different environments that are associated with situations of low immunity may be related to the predominance of infections by these gram-negative pathogens. Similar to other studies, *E. coli* was the bacteria that were most frequently found in infections in domestic animals and pets [15,20]. In research carried out with dogs and cats, *E. coli* was classified as the leading pathogen causing urinary tract diseases and the second responsible for pyodermatitis conditions [3]; however, in the present study, it was responsible for triggering a variety of infectious conditions. It is assumed that the wide range of disorders encountered can be attributed to its pathogenicity, the variety of serotypes available, and the environmental and opportunistic characteristics of this species [2].

*K. pneumoniae* was isolated in cases of pyodermatitis and in respiratory and urinary tract infections. Because it is an opportunistic bacterium, the immune status of the individual, the pathogenic capacity of the strain, and its complex antigenic structure, combined with toxin production and various resistance mechanisms, influence the severity of the infection [10,11,14]. *P. mirabilis* was identified in respiratory infections and abscesses, which corroborates different studies that indicate this bacterium as a causative agent of vaginal, uterine, respiratory, and ocular infections, as well as abscesses, sepsis, neonatal enteritis, mastitis, and dermatitis [16,21]. The combination of particularly important virulence factors, such as fimbriae, ZapA proteins, and peritrichous flagella, increases their pathogenicity to domestic animals, extending their ability to infect any organ or tissue, especially the urinary tract [22]. The isolation of *Pasteurella* spp. associated with pyometra is not frequent in the literature; however, its occurrence has been documented [23].

The high rate of antimicrobial resistance found in this study may be associated with the indiscriminate use of antibiotics, which is frequently related to the absence of medical prescriptions or the absence of previous microbiological cultures and antibiograms. In a study developed in 2011, it was also observed that the highest rates of resistance in Gram-negative bacteria were to clindamycin [3]. This high rate is possibly due to the low pharmacological activity of this drug on Gram-negative bacteria. It is believed that resistance to penicillin is represented by its indiscriminate use and short spectrum of action, acting mainly on Gram-positive bacteria [4]. The bacterial resistance presented to cephalosporins suggests the existence of resistance genes in the analyzed strains, given that these drugs are used in veterinary clinical routine in an empirical manner. In 2012, Cruz *et al*. [20] found that tetracycline was one of the most ineffective drugs against Gram-negative bacteria; the findings of our study corroborated this. The resistance to this antimicrobial probably occurs as a result of the acquisition of R plasmids, especially in enteric Gram-negative bacilli (*E. coli* and *Proteus* spp.) that are carriers of these extrachromosomal genetic elements [20]. In this study, gentamicin, although presented by other studies as a very active drug against enterobacteria [3,20], was showed low effectiveness, reinforcing the idea of the increase of bacterial resistance over the years. Regarding amoxicillin-clavulanate, it was found that only a few strains of the genus *Escherichia* showed resistance, possibly because they are the main ESBL-producing species. However, a higher percentage of sensitivity was expected since clavulanic acid associated with amoxicillin works as an inactivator of beta-lactamases [4,20]. The low effectiveness of chloramphenicol may have occurred by enzyme inactivation due to chloramphenicol-acetyltransferase, which is present in resistant bacteria [20].

The rate of multiple resistances in this study was high and was similar to that observed by Kohl *et al*. [4]. This result can be considered worrisome, as the low effectiveness of antimicrobials limits therapeutic options, with serious clinical and economic repercussions, such as increased morbidity and mortality in humans and animals [8,14,15].

A high prevalence of ESBL-producing *Enterobacteriaceae* was found. Other studies describe the high frequency of these enzymes in isolates from humans, animals, and samples of products of animal origin [6,22]. ESBL-producing *E. coli*, in this work, triggered health problems in pigs and was even more frequent in the canine species. In studies carried out in Europe, this pathogen was most commonly found in production animals and not so common in companion animals. This result can be attributed to the studies carried out by the food industry to assess the occurrence of this enzyme in production animals [6,22]. Although most of the studies focus on production animals, data on companion animals are visibly needed since ESBL-producing strains in such a group pose a potential risk to human health, either by direct transmission of resistant pathogens from animals to humans or indirectly by transmission of resistance genes [6,22]. ESBL-producing *K. pneumoniae* strains have been identified in pigs and dogs. It is important to emphasize that human beings can be colonized or infected with ESBL-producing *K. pneumoniae* by coming into contact with biological materials from carrier animals or by the consumption of contaminated water and/or food products [6].

The work conducted by Founou *et al*. [10] showed that ESBL-producing *K*. *pneumoniae* is dynamically spread in pigs and occupational workers in Cameroonian slaughterhouses. This study reinforces the need to understand the epidemiology of ESBL-producing pathogens, the transmission routes, the risk factors, and their implications for public health. In our study, samples were not collected from animal owners/caretakers to assess ESBL-positive strains; however, this procedure is indispensable due to the interspecies contact.

Two strains of *K. pneumoniae* were found to be producers of both ESBL and KPC. The dissemination of KPC in humans has been reported in several places around the world, including Brazil, the USA, China, Italy, Poland, Greece, Israel, Argentina, Colombia, and Taiwan [11]. Although there are reports on bacterial resistance in veterinary clinical isolates and in food-borne isolates, more data on the occurrence of KPC-producing *K. pneumoniae* in domestic animals are needed.

In the present study, there were no AmpC-producing samples. Such a result proves satisfactory since AmpC is produced constitutively or induced by members of the *Enterobacteriaceae* family, which hydrolyze the majority of b-lactam antimicrobials, limiting therapeutic options for treating bacterial infections [8,9,13].

## Conclusion

In this study, *E. coli* was the most frequently isolated agent, followed by *K. pneumoniae*, *P. mirabilis*, and *Pasteurella* spp. It was found that the bacterial strains presented high resistance rates to clindamycin, penicillin, imipenem, polymyxin, cefoxitin, gentamycin, cefotaxime, ceftazidime, cephalothin, ceftriaxone, ciprofloxacin, trimethoprim/sulfamethoxazole, chloramphenicol, and tetracycline. Some of the isolated *E. coli* and *K. pneumoniae* strains were identified as ESBL producers. Two strains of *K. pneumoniae* were producers of both KPC and ESBL. None of the isolates were AmpC producers. These results are concerning and signal the need for the development and implementation of measures to monitor and control the spread of highly resistant bacterial species, including performing bacterial cultures, antimicrobial sensitivity testing, and phenotypic tests to detect beta-lactam enzymes, especially in pathogenic strains.

## Authors’ Contributions

JPS and LCASV: Conception of the study. IOC: Collection and supply of the samples. JPS, VFMS, and LCASV: Conducted the experiments. JPS, VFMS, MWCS, and LCASV: Analyzed the data. JPS, JNS, NSS, APO, VTSR, and MWCS: Wrote the manuscript. LCASV and IOC: Corrected the manuscript. All authors read and approved the final manuscript.
